# The scorpion toxin and the potassium channel

**DOI:** 10.7554/eLife.00873

**Published:** 2013-05-21

**Authors:** Kenton J Swartz

**Affiliations:** 1 **Kenton J Swartz** is at the Molecular Physiology and Biophysics Section, Porter Neuroscience Research Center, National Institute of Neurological Disorders and Stroke, National Institutes of Health, Bethesda, United Statesswartzk@ninds.nih.gov

**Keywords:** ion channel, toxin, voltage-dependent K+ channel, scorpion toxin, None

## Abstract

The structure of a complex containing a toxin bound to a potassium ion channel has been solved for the first time, revealing how scorpions have designed toxins that can recognize and target the filter that controls the movement of potassium ions through these channels.

**Related research article** Banerjee A, Lee A, Campbell E, MacKinnon R. 2013. Structure of a pore-blocking toxin in complex with a eukaryotic voltage-dependent K^+^ channel. *eLife* **2**:e00594. doi: 10.7554/eLife.00594

**Image** A toxin-channel complex viewed from outside the cell; the toxin is shown in orange

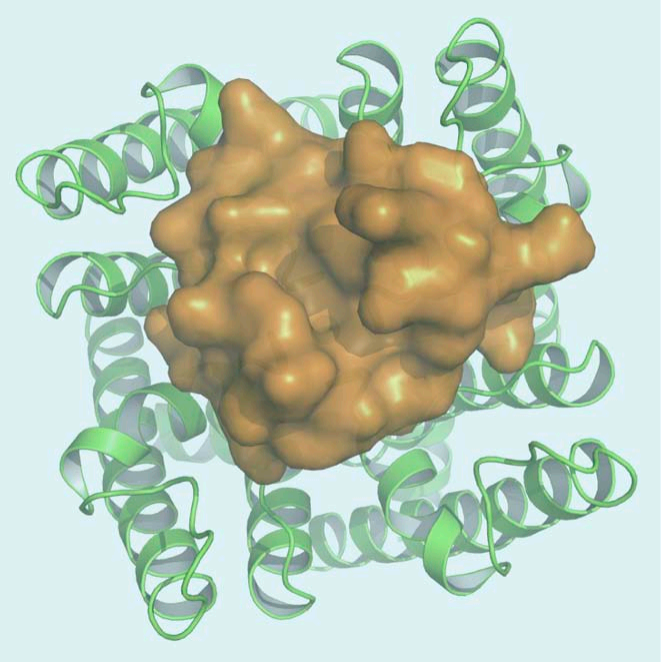

Poisonous animals such as scorpions, spiders, snakes and various marine organisms produce a bewildering array of toxins that target the nerve cells of humans and other animals. Although our understanding of these toxins and how they work remains incomplete, physiologists and pharmacologists have exploited them to explore the function of ion channels—the proteins that control the movement of ions in and out of cells. These proteins are located in the lipid membrane of the cell, and the ions enter or leave the cell via a pore that runs through the protein.

Biophysicists seeking to understand the mechanisms by which ion channels provide exquisitely selective pathways for ions to pass through biological membranes, or how voltage signals can control the opening and closing of the channels with breath taking fidelity, have used toxins as probes to identify the key structural and functional elements of these proteins (Figure 1A). Scorpion toxins that block protein pores hold a special place in the annals of ion channel biophysics and now, writing in *eLife*, Rod MacKinnon and colleagues at Rockefeller University—including Anirban Banerjee as first author—report that they have used X-ray crystallography to determine the structure of charybdotoxin, a scorpion toxin, bound to the pore of a voltage-dependent potassium ion (Kv) channel (Figure 1A; Banerjee et al., 2013).

**Figure 1.**
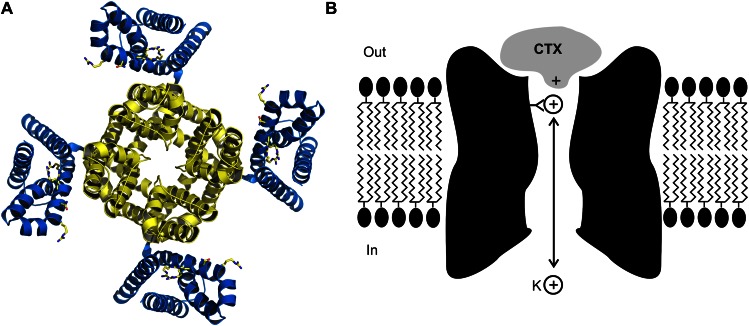
The crystal structure (**A**) of the voltage-dependent potassium ion channel studied by Banerjee et al. as viewed from outside the cell: the pore that allows the potassium ions (K^+^) to enter and leave the cell is defined by four protein subunits (shown in yellow) and is at right angles to the plane of the page. The pore domain, which is about 40 Å wide and 45 Å deep, also contains four sites within its lumen that K^+^ ions can bind to. This ion channel opens and closes in response to changes in the voltage of the lipid membrane around the cell: these changes are detected by voltage sensors (blue). Some toxins inhibit opening of the pore by binding to these voltage sensors, but charybdotoxin (not shown) targets the pore itself. (**B**) Cartoon representation of charybdotoxin (CTX) binding to the external end of a BK channel and plugging the pore (inspired by Figure 10 of MacKinnon and Miller, 1988). The outermost K^+^ ion binding site is in equilibrium with internal K^+^ ions, and when this site is occupied it repels the bound toxin.

The story begins at Brandeis University back in the 1980s, when Chris Miller and colleagues discovered that the venom of the Israeli scorpion, *Leiurus quinquestriatus*, contained a small protein that bound to a single site on the external end of a BK channel—BK is short for ‘big potassium’, and a BK channel is a particular type of Kv channel that allows a large current of potassium ions to pass through it (Miller et al., 1985). Envisioning the end of the channel as a whirlpool, they named this protein charybdotoxin after Charybdis, the daughter of Poseidon, who was turned into a whirlpool generating sea monster by Zeus. The one-to-one stoichiometry between charybdotoxin and the channel hinted that the toxin targeted the pore because it was assumed at the time that Kv channels are formed by four identical subunits and that the pore would be located at the interface between them.

MacKinnon joined the Miller lab in 1986, and together they measured how the dissociation rate of the toxin depended on the concentration of potassium ions on the inside of the cell membrane (MacKinnon and Miller, 1988). They found that increasing the internal potassium concentration caused the toxin to dissociate more rapidly, which led them to propose that the toxin bound to the external end of pore itself, as opposed to another part of the protein, and that potassium ions entering the pore from the inside could interact with the toxin (Figure 1B). In effect, this simple and elegant experiment suggested that charybdotoxin was a literal pore-blocker. When the first gene for a Kv channel was identified and sequenced (Papazian et al., 1987), Miller and MacKinnon set out to use their newly discovered pore-blocking toxin to identify the region of the channel protein that forms the outermost end of the pore. It was not long before they found that mutations within the loop between two putative membrane-spanning segments had large effects on toxin binding: this was the ground-breaking result which suggested that this region must form the external end of the pore (MacKinnon and Miller, 1989).

While much has been learned using these pore-blocking toxins in subsequent years, and X-ray structures of ion channel proteins now seem to abound, it has turned out to be exceptionally difficult to solve the structure of an ion channel with a toxin bound to it. The exceptions include the structures of snake or cone snail toxins bound to ion channels that are activated by neurotransmitter molecules (Rucktooa et al., 2009; Tsetlin et al., 2009), a tarantula toxin bound to acid-sensing ion channels (Baconguis and Gouaux, 2012; Dawson et al., 2012) and, now, the structure of charybdotoxin bound to a Kv channel (Banerjee et al., 2013).

Getting this Kv channel to crystallize with charybdotoxin bound was only the first challenge in this project. The next was finding a way to interpret the electron density maps resulting from an asymmetric toxin located on the four-fold axis of crystallographic symmetry that generates the Kv channel assembly. To help guide their model building, MacKinnon and colleagues produced three additional versions of charybdotoxin with heavy atom substitutions, showed that they remain competent to block the channel, and then crystallized each in complex with the channel.

The structure of the charybdotoxin-Kv channel complex that emerged is remarkable in several respects. First, it shows that the 27^th^ amino acid of the toxin (which is a lysine base) is found at the four-fold axis of the channel, and that this amino acid snakes its way into the pore to get tantalizingly close to the outermost of the four binding sites for potassium ions that are responsible for the potassium ion selectivity of the channel. This feature is particularly satisfying because it is precisely what MacKinnon and Miller had proposed in 1988 to explain how the potassium ions inside the cell can permeate along the pore and influence the dissociation of toxin bound to the external end of the pore (Figure 1B). It also explains why mutant toxins that do not contain a lysine base at this position are less effective at blocking this ion channel and are no longer sensitive to the concentration of internal potassium (Park and Miller, 1992).

The structures reported by Banerjee et al. are also generally consistent with results of previous experiments that identified specific amino acids in scorpion toxins that interact with amino acids within the outer pore of Kv channels, which made it possible to create a virtual map of the interaction surface between these two types of proteins (Hidalgo and MacKinnon, 1995; Naranjo and Miller, 1996). Another interesting feature of these new X-ray structures is that charybdotoxin does not alter the structure of the channel when it binds: rather, it blocks the flow of ions by fitting neatly into the pore like a cork.

With these new structures the story of the scorpion toxin and the potassium channel has come full circle. These fascinating venom toxins led the way to finding the pore before structures were available, and now we have the first images of the toxin snuggling up to the pore and placing a lysine base into the ion selectivity filter inside the pore. For the moment, the number of solved toxin-channel structures is still small, but this number is sure to increase in the future, and it will be exciting to see what they can teach us about the mechanisms of ion channel proteins.
